# *Mosla chinensis* maxim. essential oil ameliorates DSS-induced colitis and enhances the intestinal barrier via the PI3K-AKT signaling pathway

**DOI:** 10.3389/fnut.2026.1778915

**Published:** 2026-02-18

**Authors:** Jiajun Yin, Jiahao Mo, Junrong Zhu, Yifan Wu, Shiquan Lu, Jianguo Zeng, Kaijun Wang

**Affiliations:** 1 College of Veterinary Medicine, Hunan Agriculture University, Changsha, China; 2 Chinese Medicinal Materials Breeding Innovation Center of Yuelushan Laboratory, Changsha, China; 3 Xiang Ya School of Pharmaceutical Sciences, Central South University, Changsha, China; 4 Hunan Provincial Key Laboratory of the Traditional Chinese Medicine Agricultural Biogenomics, Changsha Medical University, Changsha, China

**Keywords:** gut microbiota, intestinal barrier, *Mosla chinensis* maxim., PI3K-Akt pathway, ulcerative colitis

## Abstract

Ulcerative colitis (UC) is a prevalent chronic inflammatory bowel disease characterized by recurrent episodes. *Mosla chinensis* Maxim. Shixiangru (SXR) is a traditional Chinese medicinal herb commonly utilized for treating cold, fever, diarrhea, digestive disorders, and various other ailments. And its essential oil (SEO) has been identified to exert various physiological activities. Nonetheless, there is relatively scarce information on the interaction between SEO and UC. This study investigates the anti-inflammatory properties and potential mechanisms of SEO. A DSS-induced colitis mouse model was used to investigate the anti-inflammatory effects of SEO. The DAI scores, body weight, colon length and histopathological status of colon tissue were evaluated. The levels of tight junction proteins ZO-1 and Occludin, along with inflammatory mediators including nitric oxide (NO) and cytokines such as tumor necrosis factor-*α* (TNF-α), interleukin-6 (IL-6), interleukin-10 (IL-10), and interleukin-1β (IL-1β), were analyzed. The study also investigated RNA sequencing and gut microbiota composition. Results from the DSS-induced colitis mouse model demonstrated that SEO effectively reduced inflammation and regulated gut microbiota. SEO treatment notably enhanced Tight junction (TJ) production while inhibiting the release of inflammatory mediators such as nitric oxide (NO) and cytokines including TNF-*α*, IL-6, and IL-1β. Moreover, SEO may inhibit the activation of the PI3K-AKT signaling pathway. This study highlights SEO’s efficacy in alleviating colitis and modulating gut microbiota, offering insights into its therapeutic potential for UC through anti-inflammatory effects and maintenance of intestinal homeostasis. It provides a scientific foundation for using SEO in preventing and treating inflammation and related diseases.

## 1 Introduction

Ulcerative colitis (UC) is a chronic inflammatory bowel disease (IBD) affecting the intestinal tract. The rising prevalence of UC has elevated its risk profile and has become a global problem (1). UC accompanied with symptoms including weight loss, abdominal pain and diarrhea, and may progress to colorectal cancer without intervention and control (2). It is mainly occurs in the sigmoid colon and rectum, and in severe cases can affect the entire colorectal region (3). The development of UC is multifaceted, influenced by genetic factors, environmental conditions, immune interactions, and gut microbiota (4). Currently, we are limited by our insufficient of UC prevention and treatment. Clinically, commonly used medications include amino salicylic acid (5), sulfasalazine (SASP) (6), and immunosuppressants (7) to alleviate symptoms. Nevertheless, challenges like drug resistance and significant adverse reactions frequently arise (8, 9). Therefore, there is an urgent need to discover and develop safer and efficacious drugs for UC.

Research on animal and human gut microbiota is progressing, revealing increasingly diverse functions (10, 11). The colon hosts an important and complex microbial community known as the gut microbiota, a key factor influencing human health and disease (12). This community presents thousands of bacterial species, and it plays crucial role in food digestion, nutrient absorption, bile acid metabolism, and intestinal homeostasis maintenance (13). Advancements in sequencing technology enable a more in-depth exploration of how dietary changes affect the gut microbiome in animals (14–17). Moreover, Gut microbiota can be utilized to treat inflammatory diseases through targeted modulation of specific microorganisms (18, 19). Previous study has shown that traditional Chinese herbal medicines can interact with the gut microbiota to protect the intestinal barrier (20). Despite the unclear pathogenesis of UC, substantial evidence highlights the critical role of gut microbiota in sustaining intestinal homeostasis (21). Currently, repairing intestinal barrier function by targeting specific gut bacterial strains or restoring intestinal microecological balance has become a feasible strategy for UC treatment (22). Natural products, commonly found in numerous plants and traditional Chinese herbs, demonstrate a range of biological activities such as antioxidant, antibacterial, anti-inflammatory, and anticancer effects (23). Meanwhile, Numerous natural products have been shown to a certain extent alleviate UC, such as berberine (24), curcumin (25), and andrographolide (26, 27), as a means of treatment for UC. The dextran sulfate sodium (DSS)-induced mouse model is widely used for screening potential UC (28, 29) therapeutics due to its symptomatic and pathological resemblance to human UC (30, 31).

Shixiangru (*Mosla chinensis* Maxim.), a traditional herb of the genus *Mosla* in the family Lamiaceae, is widely distributed in regions including southern China, Vietnam, India, and Japan. In China, Shixiangru has traditionally been utilized to address ailments like cold, diarrhea, abdominal pain, vomiting, and edema. Shixiangru is rich in essential oil (EOs). The essential oil (SEO) is a complex blend mainly composed of terpenes, alcohols, ketones, and other small molecules (32). Studies have reported that SEO improve human health by inhibit the early absorption process of virus strain and regulating the microbiota composition (33, 34). Leveraging the anti-inflammatory and antimicrobial properties of SEO, this study investigates its mechanism of action on UC to uncover a novel anti-inflammatory pathway. This study will provide information for the potential applications of *Mosla chinensis* Maxim. in the prevention and treatment for UC.

## 2 Materials and methods

### 2.1 Animals and materials

SPF Male C57BL/6 J mice (7–8 weeks, 20–23 g), were purchased from Guangdong Zhiyuan Biopharmaceutical Technology Co., Ltd. [Guangdong, China, license no. SCXK(YUE)2021–0057]. All mice were housed under GLP laboratory conditions and Ad libitum feeding for 7 days. Shixiangru essential oil (SEO) were provided by Hunan Phyto-way Plant Resources Co., Ltd. (Hunan, China).

### 2.2 Animals model establishment

At 8th day, the mice were randomly divided into five groups (*n* = 8), including the control group (oral administered 10 mL/kg sterile water every day for 7 days), the model group (administered 0. 9% saline every day for 7 days), the SASP group (administered 200 mg/10 mL/kg SASP for 7 days), low dose of SEO (SEO-L) group (oral administered 100 mg/10 mL/kg SEO for 7 days), and high dose of SEO (SEO-H) group (oral administered 200 mg/10 mL/kg SEO for 7 days). The doses of SEO were selected based on the effective dose ranges of congeneric plant essential oils and preliminary safety tests in our laboratory, where SEO at ≤300 mg/kg did not induce overt toxicity in mice. UC was induced in mice by administering 3.0% (w/v) DSS (Dalian Meilun Biotech Co., Ltd) in sterile drinking water for 7 days. Control mice received normal sterile drinking water throughout the experiment.

### 2.3 Collection of samples

At the end of the treatment, orbital enucleation for blood collection was performed under isoflurane anesthesia (2–3% in oxygen, inhalational) to ensure animal welfare. After blood collection, mice were euthanized via cervical dislocation while deeply anesthetized, followed by rapid dissection of colons. And blood samples were collected from each mouse using 2 mL precooled vacuum tubes. The mice were euthanized via cervical dislocation, followed by the rapid dissection of their colons. Following a previous study (35), plasma was isolated via centrifugation at 3,000 rpm for 10 min at 4 °C and subsequently stored at −80 °C for future analysis. Assessment of DAI.

### 2.4 Assessment of DAI

The disease activity index (DAI) was assessed daily throughout the experiment, based on weight loss, stool consistency, and rectal bleeding. As outlined in Table 1, weight loss is evaluated on a scale based on the percentage reduction from baseline, with scores ranging from 0 points (indicating no weight loss) to 4 points (indicating more than 20% weight loss). Stool consistency is evaluated with scores of 0 for normal, 2 for loose stool, and 4 for diarrhea, while rectal bleeding is assessed with scores of 0 for no bleeding, 2 for a positive hemoccult test, and 4 for gross bleeding. DAI scores ranging from 0 to 12 indicate the severity of colitis, from healthy to severe (36).

**Table 1:** Scoring system for caclulating DAI in mice.

Score	Body weight loss	Stool consistency	Stool hemorrhage
0	None	Normal	None
1	1–5%	Loose	
2	5–10%	watery diarrhea	Presence of blood
3	10–20%	slimy diarrhea, little blood	
4	>20%	severe watery diarrhea with blood	Gross rectal bleeding

DAI = body weight loss score + stool consistency socre + hemorrhage score.

### 2.5 Histological and colonic inflammation analysis

After evacuating the colonic content of mice, the colon tissue was rinsed with PBS and fixed in 10% buffered formalin. The samples underwent dehydration in ethanol followed by hematoxylin and eosin (HE) staining. Upon dyeing, the sections were dehydrated and mounted with neutral mounting medium. Analyzing all sections with an optical microscope. Pathological scores were evaluated following the previous study (37). As showed in Table 2, The pathological score was evaluated by summing up the scores for four histological alteration (All alteration was scored 0 to 4), including intestinal mucosal inflammation, intestinal inflammation, crypt glands, and goblet cells (38).

**Table 2:** Scoring system for DSS-induced histological changes in mice.

Score	Tissue damage	Inflammatory cell infiltration
0	None	Normal
1	None	Infrequent
2	Isolated focal epithelial damage	Increased, some neutrophils
3	Mucosal erosions and ulcers	Inflammatory cell clusters present
4	Damage deep into the colon wall	Transmural cell infiltrations

Histological score = tissue damage score + inflammatory score.

### 2.6 Enzyme-linked immunosorbent assay

Blood was collected from each mouse and centrifuged at 3000 g for 10 min at 4 °C. ZO-1 (BY-EM220420), Occludin (BY-EM220419), TNF-*α* (BY-EM220852), IL-1β (BY-EM220174), IL-6 (BY-EM220188), IL-10 (BY-EM220162) levels were determined by using ELISA kits (BOYAN, Nanjing, China) with serum and colon tissue homogenate as samples. Colon homogenates were prepared by homogenizing 50 mg of colon tissue in 500 μL RIPA lysis buffer (supplemented with 1% protease inhibitor) at 4 °C, followed by centrifugation at 12,000 × g for 15 min to collect the supernatant.

### 2.7 Determination of oxidative stress parameters and NO levels

The levels of CAT and NO were evaluated by commercial kits (Nanjing Jiancheng Bioengineering Institute Co., Ltd. Nanjing, China) following by manufacturer’s instructions. The antioxidant activity was assessed using T-AOC kits (Nanjing Jiancheng Bioengineering Institute Co., Ltd. Nanjing, China) according to the manufacturer’s instructions.

### 2.8 Gut microbiota analysis

Fecal samples from different groups of mice first stored in liquid nitrogen and subsequently kept at −80 °C. To detect structural differences in gut microbiota, we delivered samples to Beijing Biomarker Technologies Co., Ltd. for 16S rRNA sequencing. Bacterial DNA was extracted and quantified using the QuantiT™ ds DNA HS Reagent. All 16S rRNA sequencing procedures were performed by Beijing Biomarker Technologies Co., Ltd.

### 2.9 RNA-sequencing of Colon tissue

Mouse colonic tissue total RNA was extracted using the TRIzol method, and mRNA was enriched through Oligo (dT) magnetic bead selection. Libraries were prepared and sequenced using paired-end reads on the DNBSEQ-T7 platform. Post-processing identified differentially co-expressed genes, which underwent functional enrichment analysis of GO terms and KEGG pathways. All procedures were performed by Beijing Biomarker Technologies Co., Ltd. Differential expression analysis was performed using the DESeq2 package (v1.38.3) in R (v4.3.1) with thresholds of |log2 (fold change) | > 1 and adjusted *p* < 0.05. GO and KEGG pathway enrichment analyses were conducted using the clusterProfiler package (v4.6.2) in R, with adjusted *p* < 0.05 as the significance cutoff.

### 2.10 Real-time fluorescent quantitative PCR

Colon tissue total RNA was extracted using TRIZOL reagent (Solarbio Science and Technology Co., Ltd., Beijing, China). Reverse transcription was conducted utilizing the PCR kit from Accurate Biotechnology Co., Ltd., Hunan, China. cDNA was amplified using specific primers to analyze the mRNA. We utilized the Analytikjena qTOWER3G Real-Time PCR EasyTM-SYBR Green system (Germany) to evaluate target gene expression. The protocol included an initial pre-cycling step at 95 °C for 30 s, followed by 40 cycles of 5 s at 95 °C and 30 s at 60 °C. The mRNA expression levels of PI3K, AKT, Itga7, Myb, Lama3, and Ngfr in colon tissue were quantified by qPCR using SYBR Premix Ex Taq reagents (Accurate Biotechnology Co., Ltd., Hunan, China). Primer sequences are listed in Table 3, and mRNA levels were quantified using the 2−ΔΔCT method with *β*-actin as the control.

**Table 3:** Primer sequences of qRT-PCR.

Gene	GenBank accession	Primer sequences (5′–3′)
Akt1	NM_001409450. 1	Forward:5′-TGCGTGCAGAAGGAGATTGT-3′
		Reverse: 5′-GGAAGCGGTCCAGGTAGTTC-3′
PI3K	NM_001077495. 2	Forward:5′-AAACAAAGCGGAGAACCTATTGC-3′
		Reverse:5′-TAATGACGCAATGCTTGACTTCG-3′
Itga7	NM_008398. 3	Forward:5′-GTCACTCTCCCAGCCTCTCTA-3′
		Reverse:5′-AGCGCCTCTCTTCATAGGGTTC-3′
Myb	NM_010848. 3	Forward:5′-AAGGGACAGCAGGCATTACC-3′
		Reverse:5′-GGTCTGGTCCACAATGGAGG-3′
Lama3	NM_010680. 2	Forward:5′-CCAAGCAGGTCACTATGGAAATG-3′
		Reverse:5′-TCCTGTGTATCCGGGTTTGC-3′
Ngfr	NM_033217. 3	Forward:5′-ATCTTGGCTGCTGTGGTTGT-3′
		Reverse:5′-TGTCGCTGTGCAGTTTCTCT-3′
β-actin	NM_007393. 5	Forward:5′-CATCCGTAAAGACCTCTATGCCAAC-3′
		Reverse:5′-ATGGAGCCACCGATCCACA-3′

### 2.11 Statistical analysis

Data were expressed as mean ± SEM and analyzed with Graphpad Prism software version 10. A *t*-test assessed the significance of differences between two groups, whereas a one-way ANOVA with LSD multiple comparison test evaluated differences among more than two groups. Significance levels are indicated as follows: * for *p* < 0. 05, ** for *p* < 0. 01, *** for *p* < 0. 001, and **** for *p* < 0. 0001, compared to the Model.

## 3 Result

### 3.1 SEO reduces the severity of DSS-induced colitis in mice

**Figure 1:**
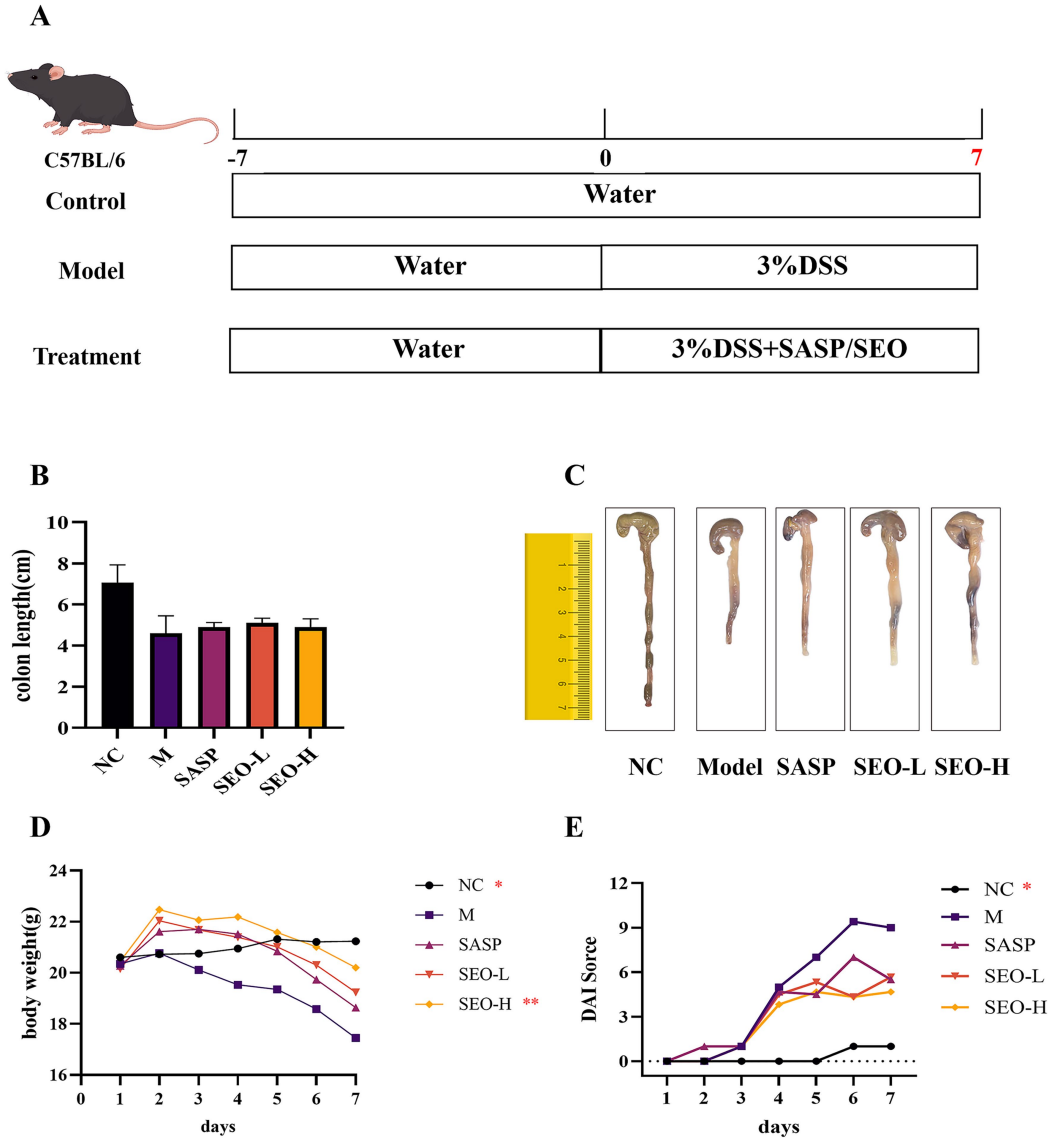
SEO ameliorated the manifestations of DSS-induced colitis. **(A)** Timeline of the animal experiment. **(B,C)** Colon length. **(D)** Body weight change. **(E)** DAI scores. The results are expressed as mean ± SD; *p < 0.05, **p < 0.01, ***p < 0.001, and ****p < 0.0001 versus the DSS group.

**Figure 2:**
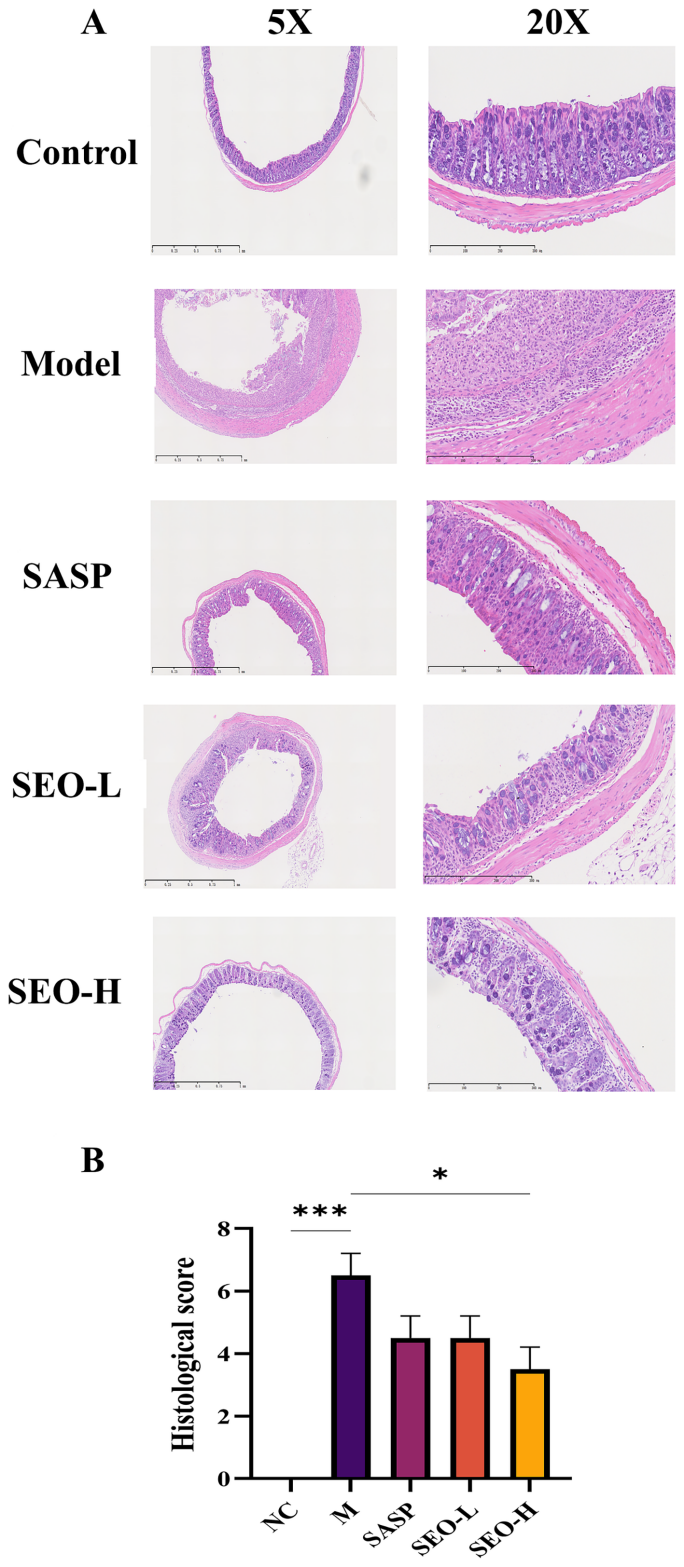
In DSS-induced mice, SEO restored the colon epithelial barrier. **(A)** A representative image of H&E-stained slices of colon tissues. **(B)** Histological scores of the tissues of the colon. The results are expressed as mean ± SD; *p < 0.05, **p < 0.01, ***p < 0.001, and ****p < 0.0001 versus the DSS group.

A 3% DSS-induced colitis model was developed to evaluate SEO’s protective effects on UC (Figure 1A). During the DSS treatment, all the mice experienced weight loss; however, we found that SEO greatly alleviated this loss (Figure 1D). Compared with the DSS group, mice in the SEO plus DSS group exhibited significantly lower DAI scores and longer colons after repeated cycles of the DSS treatment that in the DSS group did, suggesting less inflammation (Figures 1B–E). H&E staining and histological analysis indicated that SEO treatment mitigated DSS-induced tissue damage and inflammatory cell infiltration in the mucosa and submucosa, resulting in a reduced microscopic histological score compared to the DSS group (Figures 2A,B). The data suggested that SEO may have a protective effect against DSS-induced colitis.

### 3.2 SEO alleviated inflammation caused by DSS-induced UC

**Figure 3:**
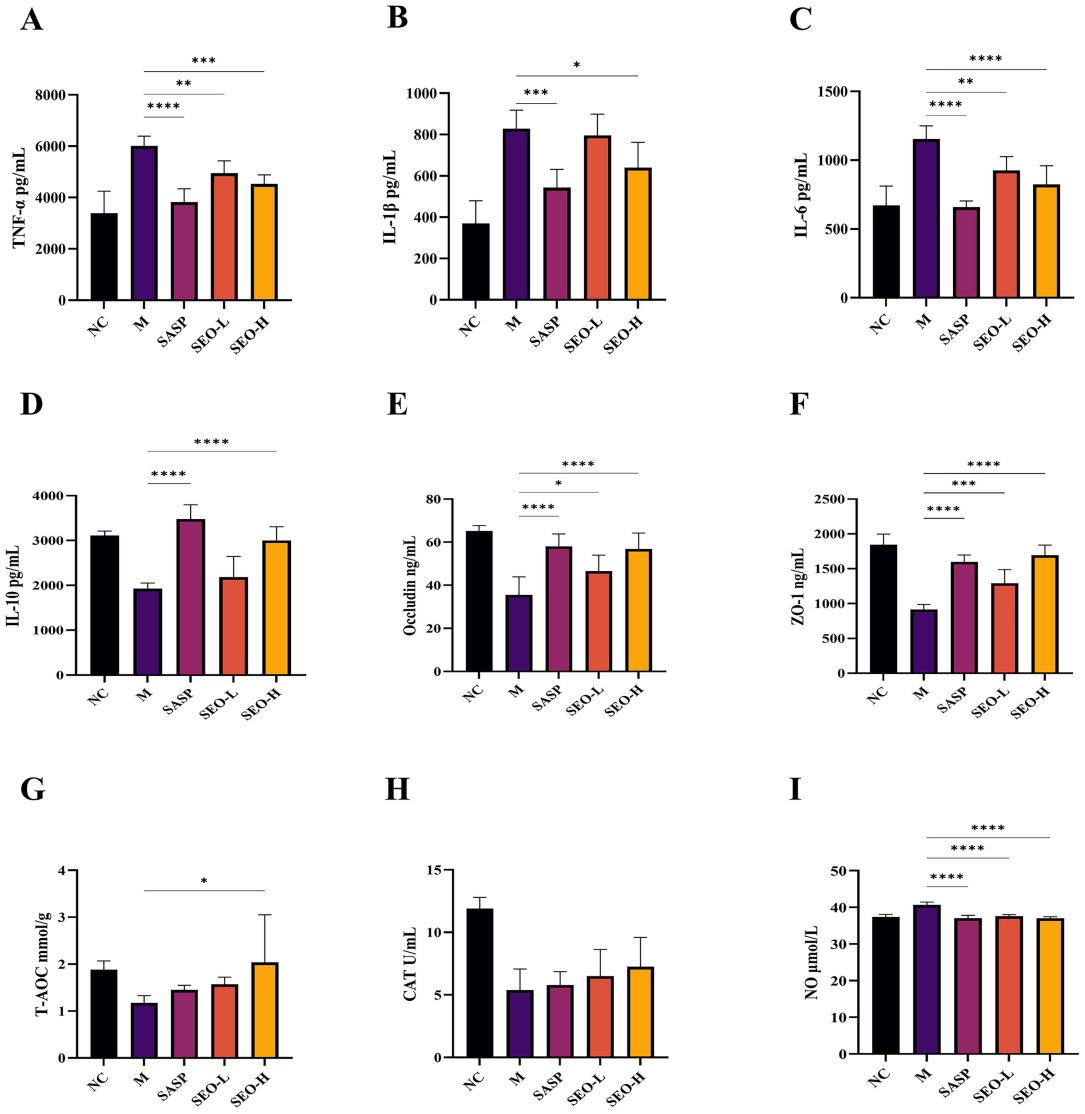
Effect of SXR on the content of TNF-*α* **(A)**, IL-1*β* **(B)**, IL-6 **(C)**, IL-10 **(D)**, Occludin **(E)**, ZO-1 **(F)** in colon tissues. The effect of SXR on the content of T-AOC **(G)**, CAT **(H)**, NO **(I)** in serum. The results are expressed as mean ± SD; *p < 0.05, **p < 0.01, ***p < 0.001, and ****p < 0.0001 versus the DSS group.

To assess the effect of SEO on inflammatory symptoms, we detected the levels of several key molecules. The findings showed that SEO mitigated the DSS-induced increase in TNF-*α*, IL-1β, IL-6, and IL-10 expression (Figures 3A–D). Meanwhile, high dose SEO showed more effective inhibitory impact. We also found that SEO increased the levels of T-AOC and CAT, while decreasing the level of NO (Figures 3G–I). The findings indicate that SEO has potent anti-inflammatory effects by modulating the expression levels of molecules involved in pro-inflammatory cytokine production.

### 3.3 SEO maintains the integrity of the intestinal barrier

A hallmark change in UC is the compromised integrity of the intestinal barrier. Tight junction (TJ) proteins are crucial for bridging intercellular gaps and regulating intestinal mucosa permeability. Previous studies have shown that TJ protein levels are inversely related to the severity of UC. ZO and claudin family proteins collectively form tight junction (TJ) proteins. Inflammatory factors such as IL-6, IL-1β, and TNF-*α* decrease TJ protein levels, resulting in increased intestinal permeability and impaired barrier function. To confirm this, we assessed the levels of ZO-1 and Occludin (Figures 3E,F). These results suggested that SEO can maintain intestinal barrier integrity by modulating the levels of ZO-1 and Occludin to preserve TJ homeostasis.

### 3.4 SEO changes the composition of the gut microbiota

**Figure 4:**
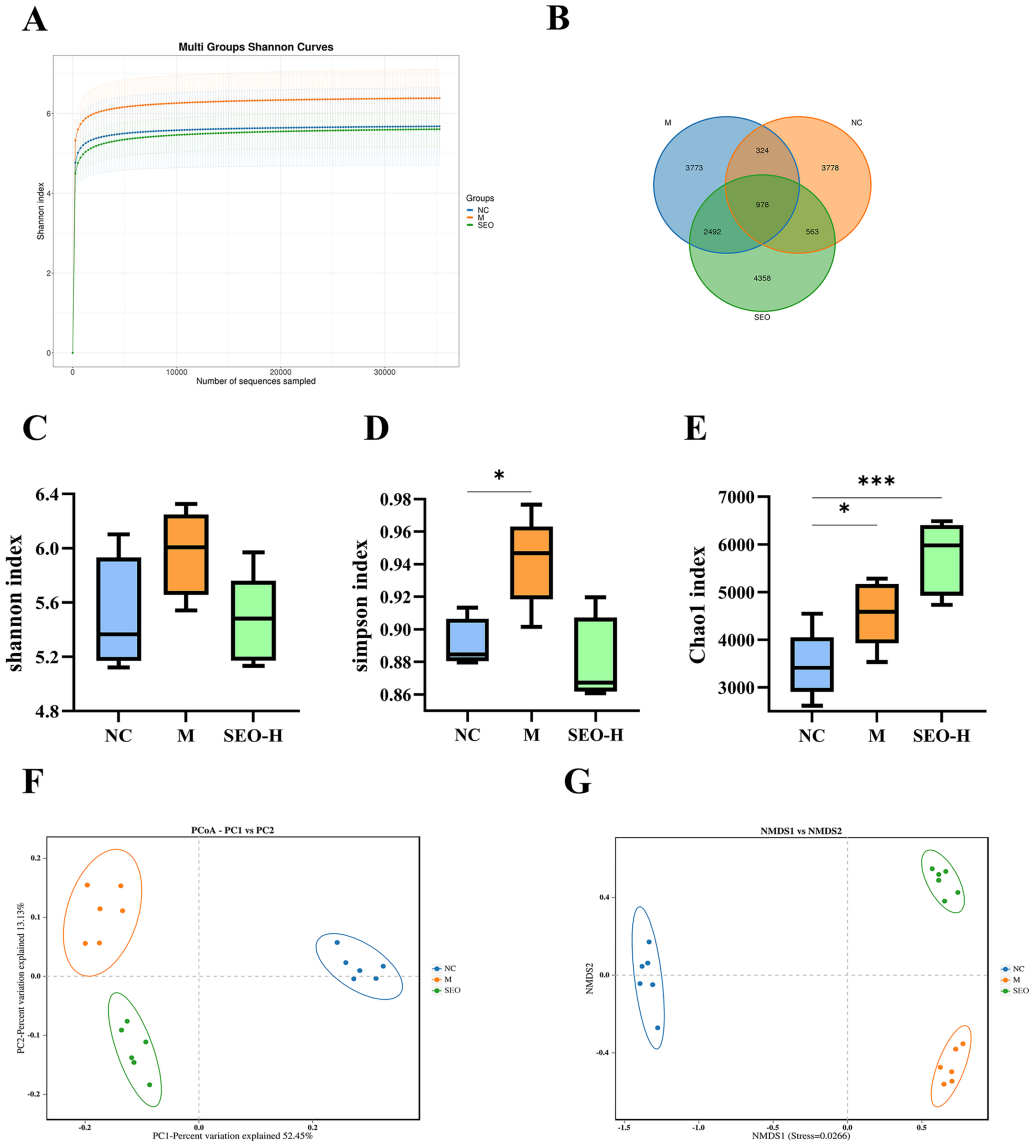
16S rRNA analysis of microbiota in the colonic content at the diversity. **(A)** Shannon index curve. **(B)** Venn diagram showed common species comparison with the three groups. **(C)** Shannon index analysis. **(D) S**impson index analysis. **(E)** Chao1 index analysis. **(F)** PCoA analysis **(G)** NMDS analysis. *p < 0.05, **p < 0.01, ***p < 0.001, and ****p < 0.0001 versus the DSS group.

Intestinal dysbiosis is one of the important pathogenic factors in the development of UC. We utilized 16S rRNA sequencing to explore the anti-inflammatory effects of SEO and its influence on gut microbiota composition. The rarefaction curves in the alpha diversity analysis plateaued, suggesting that the sequencing depth adequately captured the sample diversity (Figure 4A). The Wayne plot results revealed that the model group, DSS + SEO-H (200 mg/10 mL/kg) group, and control group identified 3,773, 4,358, and 3,778 independent OTUs, respectively (Figure 4B). We evaluated the alpha diversity index to determine the effect of SEO on the abundance and diversity of intestinal flora. The results indicated that the model group had higher Shannon and Simpson indices, while the SEO-H group showed a significant decrease in these indices. Furthermore, the SEO group increased in Chao 1. Consequently, SEO may enhance the richness and diversity of intestinal microbiota (Figures 4C–E). Beta diversity indices, including PCoA and NMDS, were employed to assess the similarities and differences among the flora. Both indices showed notable alterations in the intestinal flora of DSS-induced mice, which were alleviated by SEO (Figures 4F,G). The findings indicated that SEO influenced the composition of intestinal flora.

**Figure 5:**
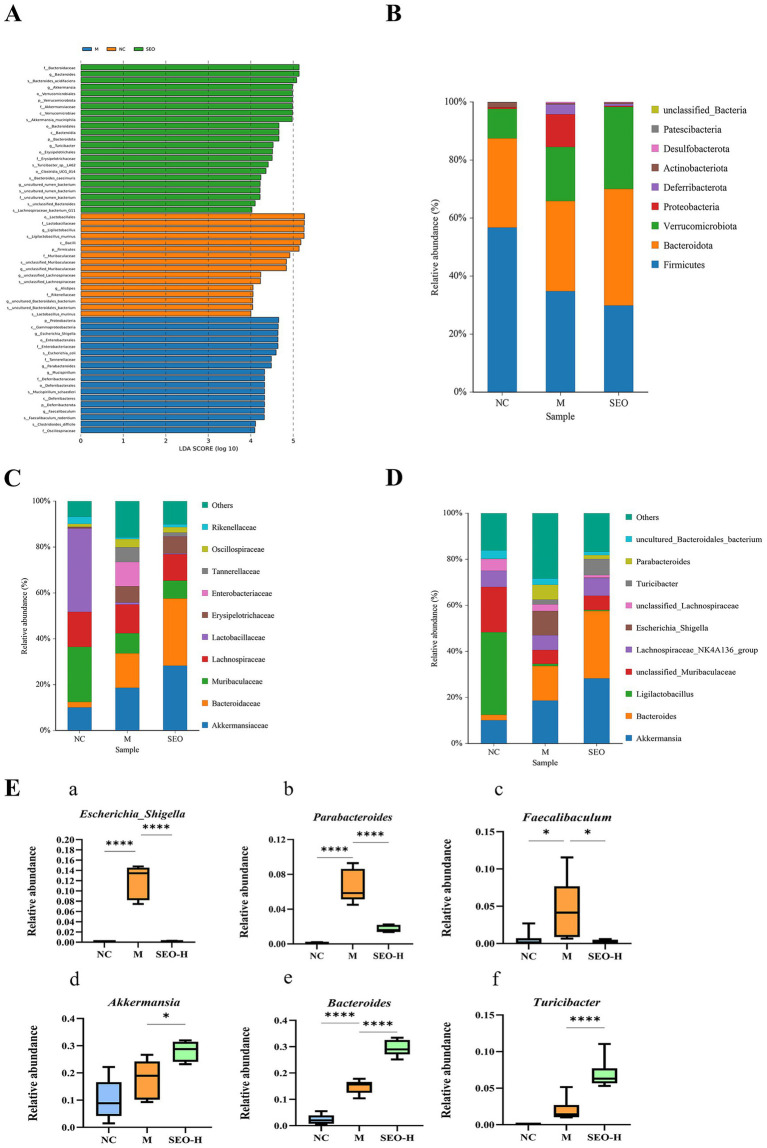
Comparisons of gut microbial compositions. **(A)** Taxa meeting an LDA score threshold > 2 are shown. **(B–D)** Composition at the phylum, family, and genus levels. **(E)** The quantity and makeup of the intestinal flora in the colon at the genus level within each group. The relative abundance of *Escherichia_Shigella* **(a)**, *Parabacteroides* **(b)**, *Faecalibaculum* **(c)**, *Akkermansia* **(d)**, *Bacteroides* **(E)**, and *Turicibacter* **(F)** were caculated. The results are expressed as mean ± SD; *p < 0.05, **p < 0.01, ***p < 0.001, and ****p < 0.0001 versus the DSS group.

The impact of SEO administration on intestinal microbial composition was assessed using linear discriminant analysis (LDA) effect size (LEfSe), following an analysis of intestinal flora diversity (Figure 5A). The study found that *Escherichia_Shigella*, *Parabacteroides*, *Mucispirillum*, and *Faecalibaculum* were the dominant bacterial genera in the mouse model group. *Bacteroides* and *Akkermansia_muciniphila* predominated in the SEO-H group.

We analyzed changes in gut microbiota composition at the phylum, family, and genus levels, as depicted in Figures 5B–D, respectively. SEO significantly decreased the relative abundance of *Proteobacteria*, *Deferribacterota*, *Enterobacteriaceae*, and *Clostridiaceae*, while increasing *Bacteroidota*, *Verrucomicrobiota*, and *Akkermansiaceae* in DSS-treated mice. SEO decreased the relative abundance of the genera *Escherichia_Shigella*, *Parabacteroides*, and *Faecalibaculum*, while it increased the abundance of *Akkermansia*, Bacteroides, and *Turicibacter* (Figure 5E). Overall, SEO alleviated UC by regulating specific gut microbiota.

### 3.5 SEO protect the colon via the PI3K-AKT pathway

**Figure 6:**
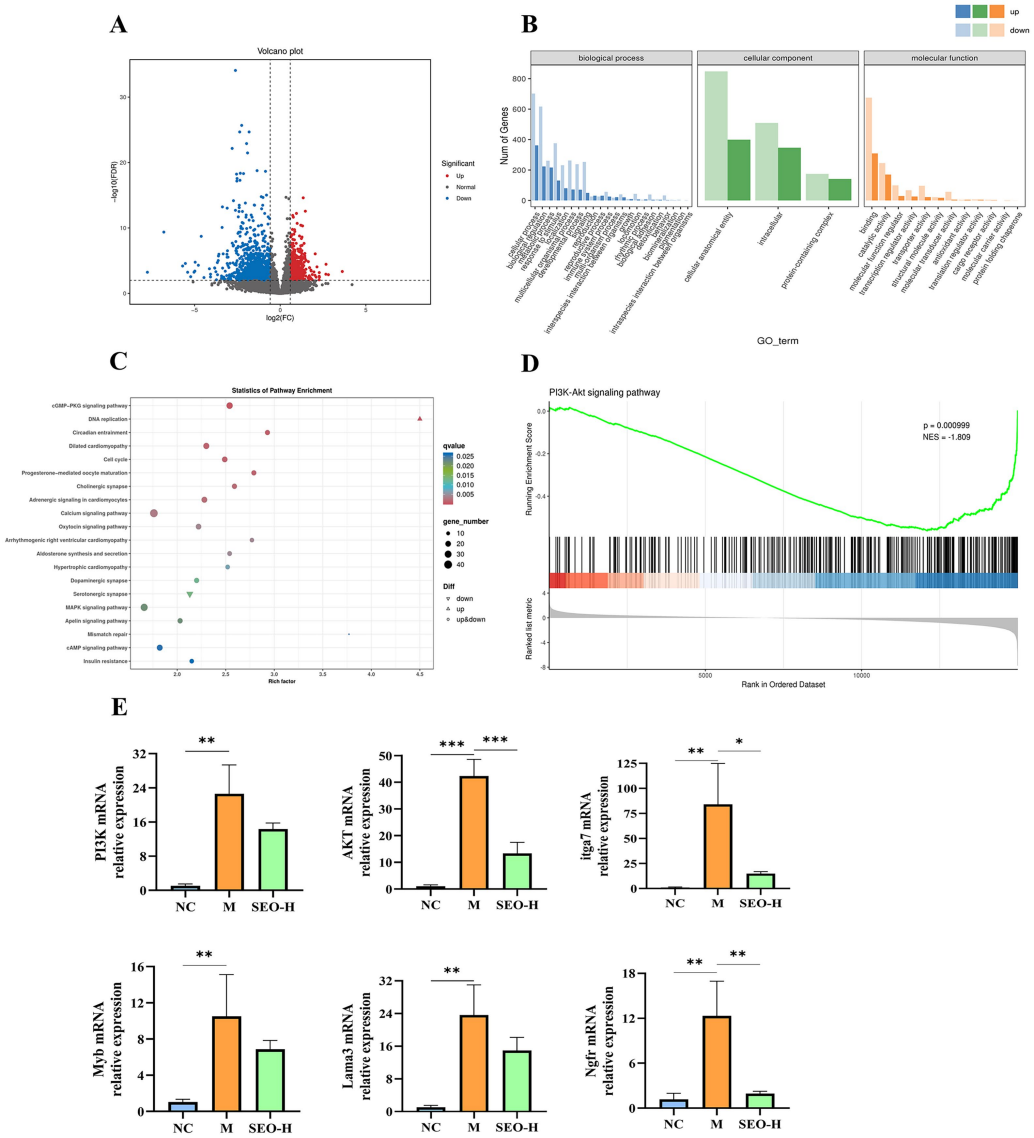
RNA-seq of colon tissues. **(A)** Volcano plot of differentially expressed genes between DSS group and SEO-H group. **(B)** The results of GO enrichment analysis. **(C)** The outcome of KEGG pathway enrichment analysis of DEGs. **(D)** Results of GSEA analysis of the PI3K-AKT pathway in DEGs. **(E)** The results of PI3K-AKT signaling pathway related genes mRNA expression of mice in different groups. The results are expressed as mean ± SD; *p < 0.05, **p < 0.01, ***p < 0.001, and ****p < 0.0001 versus the DSS group.

To elucidate how SEO mitigates ulcerative colitis, we utilized RNA-seq to investigate the biological functions and molecular mechanisms of differentially expressed genes (DEGs) through integrated analysis. Transcriptome sequencing revealed 1,666 differentially expressed genes (DEGs) in the volcano plot, comprising 515 up-regulated and 1,151 down-regulated DEGs (Figure 6A). Gene Ontology (GO) annotation of all differentially expressed genes (DEGs) identified several biological processes (BP), primarily encompassing cellular processes, biological regulation, metabolic processes, and response to stimuli. The differentially expressed genes (DEGs) showed enrichment in cellular components such as cellular anatomical entities, intracellular structures, and protein-containing complexes. The molecular functions (MC) related to DEGs were mainly binding and catalytic activities. (Figure 6B). Furthermore, KEGG pathway enrichment analysis identified signaling pathways associated with the DEGs. Consequently, several significant KEGG signaling pathways were identified, as illustrated in Figure 6C. KEGG pathway enrichment analysis and GSEA of RNA-seq-derived DEGs identified the PI3K-AKT pathway as notably prominent (Figure 6D). The PI3K-AKT pathway is linked to cell differentiation and proliferation, and it can modulate various classic inflammatory immune-related pathways, either activating or inhibiting them (39). qPCR assays were performed to determine if SEO protected colon tissues by affecting the PI3K-AKT pathway.

In DSS-induced mice, the expression levels of PI3K, AKT, Itga7, Lama3, and Ngfr genes were significantly elevated compared to the control group. However, these levels were notably reduced in the SEO-H group relative to the model group (Figure 6E). This could be due to the inhibition of the PI3K-AKT pathway. These findings further confirm that SEO protect the DSS-induced in mice via the PI3K-AKT pathway.

## 4 Discussion

Ulcerative colitis is a persistent, unexplained, and non-curable inflammatory bowel disease characterized by frequent relapses, posing a significant global health challenge (40). Issues such as steroid dependence, adverse reactions, and drug resistance commonly associated with first-line treatments need to be resolved. Consequently, it is imperative to explore new and effective treatments.

DSS-induced UC models replicate clinical and pathological characteristics of human UC, including weight loss, diarrhea, hematochezia, diffuse colonic inflammation, and intestinal barrier damage. These models have thus become a valuable tool for investigating UC pathogenesis and evaluating potential therapeutic agents (36, 38). A DSS-induced colitis mouse model was used in our study to assess the impact of SEO on UC. SEO-H treatment increased body weight, reduced DAI resource, and ameliorated multiple pathological injuries, including colonic mucosal integrity destruction, inflammatory cell infiltration, and crypt damage. The SEO-H group exhibited increased protein expression of ZO-1 and Occludin compared to the model group. This safeguards the intestinal barrier’s integrity, preventing microbial translocation and inflammatory substance leakage (41). Therefore, SEO-H effectively alleviated the intestinal barrier lesions and inflammatory symptoms induced by DSS.

Increasing evidence indicates that inflammation and intestinal barrier dysfunction are central to the pathology of UC and are vital targets for understanding its pathogenesis and developing treatments. According to references (42–44), the formulation and composition of a diet can influence animal growth performance and antioxidant capacity. Our research confirms that the addition of high doses of SEO increases the antioxidant capacity of animals. Anti-inflammatory and immunomodulatory properties, alongside antioxidant capacity, are crucial for host well-being (45). Cytokines, released by activated immune cells, are a class of biologically active substances. Immune cells interact and regulate each other, playing vital roles in inflammation, immune responses, tissue healing, and hematopoiesis (46–48). The intestinal epithelium, the body’s largest interface with the external environment, serves as a crucial barrier via the mucosa, selectively restricting toxin and antigen penetration while facilitating nutrient and water absorption (49). Dietary nutrients can influence the structure of small intestinal tissue and animal digestive function (50, 51). Disruption of intestinal mucosal homeostasis can occur due to an imbalance between gut microbiota and other factors (52). The intestinal epithelial barrier serves as the primary defense between the luminal environment and the host, and its compromise can lead to severe inflammation or intestinal diseases (53). Tight junctions (TJs) are a critical physical barrier in the gut, with their structure and function primarily reliant on transmembrane proteins like Occludin and scaffold proteins such as ZO-1. These components are essential for maintaining intestinal barrier homeostasis and regulating permeability (54, 55). Under pathological conditions, the substantial loss of tight junctions, such as ZO-1 and Occludin, can compromise intestinal barrier function and trigger inflammatory responses (56). Knockdown of ZO-1 or Occludin in mice has been shown to elevate the risk of gut inflammation and impede mucosal repair, suggesting the involvement of the intestinal barrier in the onset and progression of UC (57). The interaction between the intestinal barrier and immunity is crucial in the development of UC. Disruption of the intestinal barrier can trigger aggressive immune responses, resulting in the excessive release of pro-inflammatory factors such as IL-1β, IL-6, and TNF-*α*, which exacerbate colon injury (58). In ulcerative colitis (UC), severe inflammation in colon tissue is strongly inversely related to intestinal barrier function, elevating the risk of colorectal cancer (CRC) (59, 60). Inhibiting gut inflammation effectively safeguards the intestinal barrier and reduces the risk of colorectal cancer. Our research demonstrated that SEO treatment significantly suppresses the expression of proinflammatory cytokines IL-1β, IL-6, and TNF-α in UC mice and intestinal organoids. Furthermore, SEO prevented the DSS-induced rise in intestinal permeability and maintained intestinal barrier integrity. The findings suggest that SEO’s protective effect in alleviating UC is likely linked to its ability to inhibit inflammation and preserve intestinal barrier integrity.

The gut microbiota is essential for host homeostasis, influencing immunity, metabolism, and signaling pathways (61–63), and its imbalance is linked to the development and progression of UC (64, 65). In our study, we found SEO intervention favorably modulates this dysbiosis. Specifically, SEO treatment reduced the abundance of the pro-inflammatory, adherent-invasive *Escherichia_Shigella* (66, 67) while concomitantly enriching *Akkermansia*. The increase in *Akkermansia* is particularly noteworthy. Although its role can be context-dependent, with one report suggesting pathogenicity in genetically susceptible hosts (68), the predominant evidence highlights its function in strengthening the mucosal barrier and promoting epithelial repair, thereby protecting against colitis (69, 70). Therefore, based on phenotypic assessments such as the histopathological scores, SEO treatment may associate with a suppression of pathogenic microbiota and an increase in beneficial microbiota.

While prior research has demonstrated that SEO alleviates inflammation and enhances gut microbiota in mice with DSS-induced colitis through the MAPK signaling pathway (34), the precise molecular mechanism of SEO in colitis is still not fully understood. To explore its protective mechanism, RNA-seq analysis was performed. Post-DSS administration, there was a notable upregulation of genes associated with the PI3K-AKT signaling pathway. AKT, a key component of the PI3K-AKT signaling pathway, is crucial for various normal cellular functions. Conversely, its dysregulation is fundamental to several pathological conditions, such as inflammatory and autoimmune diseases, overgrowth syndromes, and neoplastic transformation (71). Ngfr (nerve growth factor receptor) activates signaling pathways like NF-κB, which promotes the release of pro-inflammatory cytokines such as TNF-*α*, IL-6, and IL-1β, thus enhancing the inflammatory response. The SEO treatment group significantly decreased the mRNA expression levels of PI3K-AKT pathway-related genes, such as AKT, Ngfr, and Itga7, compared to the model group. The study found that SEO suppressed the activation of the PI3K-AKT pathway induced by DSS.

## 5 Conclusion

In summary, the findings indicated that SEO alleviated DSS-induced effects in mice, mitigated intestinal mucosal barrier damage, and enhanced ZO-1 and occludin protein expression. Importantly, SEO treatment decreased the gene expression of PI3K-AKT signaling, such as PI3K, AKT, Itga7, Myb, Lama 3, and Ngfr. Meanwhile, SEO increased the relative abundance of *Bacteroides* and *Akkermansia_muciniphila* while suppressing *Escherichia_Shigella*, *Faecalibaculum*, *Mucispirillum*, and *Clostridiodes*. The specific compound within the SEO mixture responsible for the most effective anti-inflammatory effects and its metabolic behavior *in vivo* remain unclear. Future research could explore SEO extracts by examining the upstream and downstream signals of the PI3K-AKT pathway and investigating its anti-inflammatory mechanisms using gene silencing or knockdown experiments. These findings offer insights into the anti-inflammatory effects of traditional Chinese medicine SEO in DSS-induced colitis mice, serving as a reference for its potential use in preventing or treating inflammation and related diseases.

## Data Availability

The 16s rRNA sequencing data has been released at the National Genomics Data Center (https://ngdc.cncb.ac.cn/), numbered PRJCA057263.
